# Ras Homolog A (RhoA) Is Involved in the Innate Immune Defense of the Red Swamp Crayfish *Procambarus clarkii*

**DOI:** 10.3390/biology15020112

**Published:** 2026-01-06

**Authors:** Shengjie Ren, Wenjing Xu, Xianjun Ma, Chunhua Ma, Aimin Wang, Qiuning Liu, Lishang Dai

**Affiliations:** 1 State Key Laboratory Incubation Base for Conservation and Utilization of Bio-Resource in Tarim Basin, Tarim University, Alar 843300, China; 2 Yancheng Institute of Technology, College of Marine and Bioengineering, Yancheng 224000, China; 3 Alar City Gudukou Aquaculture Farmers Professional Cooperative, Alar 843300, China; 4 School of Wetlands, Yancheng Teachers University, Yancheng 224007, China; 5 School of Pharmaceutical Sciences, Wenzhou Medical University, Wenzhou 325035, China

**Keywords:** *Procambarus clarkii*, RhoA, RNAi, gene expression, immune response

## Abstract

RhoA (Ras homolog A) is a prominent member of the Rho GTPase family, playing a key role in various cellular processes such as cytoskeletal dynamics, cell migration, and immune responses. However, its function in red swamp crayfish remains unclear. In this study, it is proposed that RhoA may regulate the innate immune response in *P. clarkii*. The gene was fully characterized as *PcRhoA* in *P. clarkii*. The results showed that the open reading frame (ORF) of *PcRhoA* is 663 bp, encoding a 220-amino acid protein with a conserved Rho domain of 174 amino acids. Phylogenetic analysis placed PcRhoA close to *Cherax quadricarinatus* RhoA. RT-qPCR analysis revealed high expression levels of the *PcRhoA* gene in the hepatopancreas, muscle, heart, ovary, and stomach, with lower expression in the blood, intestine, gills, and tentacle gland. Furthermore, *PcRhoA* mRNA transcript was significantly upregulated in the intestine following LPS and Poly I:C challenges. Knockdown of *PcRhoA* suppressed the expression of downstream genes in the immune signaling pathway. These results indicate that *PcRhoA* appears to play a pivotal role in regulating the immune response of crayfish.

## Simple Summary

The *RhoA* gene was identified in red swamp crayfish, where it functions as a key regulator of the animal’s immune defenses. Following immune challenges, the *PcRhoA* gene’s activity increases in the intestine, and silencing it impairs immune gene expression. These findings highlight *RhoA*’s critical role in crayfish immunity and its potential to support healthier breeding practices without the need for antibiotics.

## 1. Introduction

*Procambarus clarkii* (Crustacea: Decapoda: Cambaridae), commonly known as the red swamp crayfish, is native to the southern and southeastern United States and northern Mexico [1]. Due to its well-balanced amino acid composition, high vitamin content, and exceptional palatability, *P. clarkii* has become one of China’s most economically significant aquaculture species, supporting a multi-billion-yuan industry [2]. However, rapid industrial expansion has led to the introduction of numerous toxicants—including pesticides, fertilizers, and heavy metals—into aquatic ecosystems, threatening the water quality essential for crayfish farming. Additionally, devastating epizootics caused by parasites, bacteria (*Vibrio parahaemolyticus*, and *Vibrio harveyi*), and viruses (white spot syndrome virus) have repeatedly ravaged crayfish farms, resulting in substantial economic losses. Understanding the innate immune system of *P. clarkii* is therefore critical for advancing knowledge of invertebrate immunity and ensuring the long-term sustainability of the crayfish aquaculture industry [3]. The crayfish intestine, a tubular organ comprising the midgut and hindgut, is responsible for digestion, nutrient absorption, and immune surveillance. Its epithelial surface is covered by a mucus layer that is densely populated with a commensal microbiota, which aids in feed breakdown and pathogen exclusion. Consequently, shifts in intestinal microbial composition—caused by farming practices, water quality changes, or xenobiotic exposure—serve as a sensitive biomarker of host health and environmental stress [4].

The immune system serves as a critical defense mechanism that enables organisms to recognize and eliminate invading pathogens. It is broadly categorized into two systems based on specificity and memory: innate immunity and adaptive immunity [5]. Innate immunity, evolutionarily conserved across both invertebrates and vertebrates, represents the primary defense in crustaceans such as *P. clarkii* [6,7,8]. This defense operates through three interconnected strategies: physical barriers, including the cuticle and exoskeleton, which prevent pathogen entry; humoral responses, involving the secretion of soluble immune effectors like lysozymes and antimicrobial peptides (AMPs) into the hemolymph to directly kill or inhibit microbial growth; and cellular immunity, where hemocytes mediate phagocytosis, encapsulation, and nodulation to clear invading microorganisms [9,10,11]. Key to these processes are the Toll, IMD (immune deficiency), and JAK/STAT signaling pathways, which drive rapid and effective innate immune responses in invertebrates [7,10,11,12].

The small GTPase superfamily is a crucial group of intracellular signaling proteins that regulate cytoskeletal dynamics, cell migration, proliferation, and immune defense [13]. RhoA, a prototypical member of this family, has been extensively studied in mammals, where it modulates various biological processes by regulating actin dynamics and downstream signaling pathways [14]. Beyond cytoskeletal regulation, RhoA governs immune cell activation, chemotaxis, and phagocytosis—functions vital for pathogen resistance, particularly during bacterial infections [15]. In aquatic species, RhoA acts as a central signaling hub, linking immune perception to effector responses through its molecular switch activity, coordinating actin reorganization, signal transduction, and transcriptional reprogramming [16]. RhoA integrates multiple immune signaling pathways, including Toll-like receptor (TLR), nuclear factor-κB (NF-κB), and mitogen-activated protein kinase (MAPK) modules, forming a complex network that ensures timely and robust immune responses [17]. Despite advances in understanding RhoA’s functions, its biological roles and signaling mechanisms in aquatic animals, especially crustaceans, remain poorly defined. In this study, *RhoA*, a member of the Rho GTPase family, was identified through transcriptome sequencing of *P. clarkii* (denoted as *PcRhoA*). Comparative analysis of RhoA protein sequences from various species revealed their phylogenetic relationships. *PcRhoA* was expressed across different tissues. After *RhoA* silencing and immune challenges, the expression levels of genes involved in AMP synthesis were analyzed. These findings offer new insights into the immune role of *RhoA* in *P. clarkii*.

## 2. Materials and Methods

### 2.1. P. clarkii and Tissue Collection

*P. clarkii* specimens were obtained from an aquatic market in Yancheng, Jiangsu Province, China, with individual weights ranging from 20 to 25 g. Prior to the experimental procedures, these crayfish were acclimatized in a laboratory incubator, supplied continuously with fresh water at a controlled temperature of 24 °C for two weeks [18]. Following the acclimatization period, dissection was performed to collect various tissues, including the hepatopancreas, muscles, intestines, gills, hearts, stomachs, ovaries, spermaries, brains, ventral nerve cords, and antennal glands. Approximately 200 µL of hemolymph was sampled from each crayfish (*n* = 5 per experimental group) via puncture at the base of a walking leg using a sterile, ice-chilled syringe. Immediately upon extraction, each 200 µL hemolymph sample was mixed with an equal volume (200 µL) of ice-cold, sterile anticoagulant solution. The anticoagulant used was modified Alsever’s solution (final concentrations: 27 mM sodium citrate, 336 mM NaCl, 115 mM glucose, 9 mM EDTA, pH 7.4), as cited from our reference [19]. This 1:1 dilution was performed directly in a pre-chilled microcentrifuge tube to instantly inhibit coagulation and melanization. The diluted hemolymph was then immediately subjected to centrifugation at 2000 rpm for 10 min at 4 °C to isolate the hemocyte pellet for downstream RNA extraction. The entire process—from hemolymph draw to the start of centrifugation—was completed within 2–3 min per individual to ensure sample integrity.

### 2.2. Immunity Challenge

The 60 crayfish were randomly divided into three groups, with each group receiving an injection of 100 µL of either lipopolysaccharide (LPS) or Poly I:C to induce an immune response, while the control group was injected with an equivalent volume of phosphate-buffered saline (PBS) [20]. Five crayfish were randomly selected from each group, and intestines were sampled for real-time quantitative PCR (RT-qPCR) analysis at 12, 24, 36, and 48 h post-injection (hpi) [21].

### 2.3. RNA Extraction and cDNA Synthesis

Total RNA was extracted from the tissues of crayfish in both control and challenge groups at different time points using the RNA Isolater Total RNA Extraction Reagent (Vazyme, Nanjing, China). The RNA was reconstituted in DEPC-treated water, and its quality was assessed by 1% agarose gel electrophoresis, ensuring no RNase contamination. The first strand of complementary DNA (cDNA) was synthesized using a cDNA Synthesis Kit (Vazyme, Nanjing, China), and the cDNA samples were stored at −80 °C for future analysis.

### 2.4. Cloning of the PcRhoA Gene

Prior transcriptomic analyses have identified the specific forward and reverse primers for the *PcRhoA* gene [8,18]. The cloning of the open reading frame (ORF) of *PcRhoA* was achieved using the 2X PCR Mix (Vazyme Nanjing, China) with the following PCR protocol: an initial denaturation step at 95 °C for 3 min, followed by 35 amplification cycles of 95 °C for 20 s, 55 °C for 25 s, and 72 °C for 30 s, ending with a final extension at 72 °C for 10 min. The resulting PCR products were purified using a Vazyme purification kit (Nanjing, China), then inserted into a pMD-19T vector (Takara, Dalian, China) and introduced into competent *Escherichia coli* (DH5α) cells. Positive recombinant clones were identified using blue-white color selection on ampicillin-containing LB agar plates, along with PCR screening using two distinct primers. Confirmation of positive clones was performed through sequencing by Sangon Biotech (Shanghai, China).

### 2.5. Sequence Blast and Phylogenic Analysis for the PcRhoA Gene

The Expert Protein Analysis System, available at http://www.expasy.org (accessed on 15 May 2024), was used to retrieve deduced amino acid sequences. These sequences were then evaluated using the ORF identification tool at http://ncbi.nlm.nih.gov/gorf/gorf.html (accessed on 20 May 2024). Protein domain predictions were made using the Simple Modular Architecture Research Tool (SMART) program (http://smart.embl-heidelberg.de (accessed on 21 May 2024)). To obtain a dataset suitable for phylogenetic analysis, homologous RhoA protein sequences from diverse species were first compiled. Multiple sequence alignment was then performed on these sequences using Clustal X software (https://evomics.org/resources/software/bioinformatics-software/clustal-x/ (accessed on 22 May 2024)) with default parameters. The resulting alignment was visually inspected for conserved regions and overall quality to confirm its appropriateness before proceeding to tree construction [22]. The phylogenetic tree was generated using Molecular Evolutionary Genetics Analysis (MEGA) version 12 [23].

### 2.6. RT-qPCR Analysis for Expression Patterns

cDNA was synthesized from RNA extracted from various tissues of both unchallenged and challenged crayfish at different time points. Gene-specific primers were designed for this purpose, and the expression of *Pc18S* was used as an internal control (Table 1). RT-qPCR was performed in a 10 μL reaction volume containing 5 μL of qPCR mix, 2 μL of cDNA diluted 1:9, 0.5 μL of each forward and reverse primer, and 2 μL of double-distilled water (ddH_2_O). The amplification protocol included an initial denaturation at 95 °C for 2 min, followed by 40 cycles of denaturation at 95 °C for 15 s, annealing at 56 °C for 20 s, and extension at 72 °C for 30 s. A melting curve analysis was conducted between 60 °C and 95 °C. Relative gene expression levels were calculated using the 2^−ΔΔCT^ method [24].

### 2.7. Double-Stranded RNA Synthesis and RNAi Assay

Double-stranded RNA (dsRNA) targeting either the *PcRhoA* gene or green fluorescent protein (GFP) as a control was synthesized using an in vitro transcription T7 kit (Vazyme, Nanjing, China). To generate the DNA template for the synthesis of PcRhoA-dsRNA or GFP-dsRNA, PCR was performed with primers, ds-PcRhoA-F and ds-PcRhoA-R, or ds-GFP-F and ds-GFP-R (Table 1). The dsRNA synthesis reaction was carried out in a total volume of 20 μL, comprising 8 μg of DNA template, 2 μL of transcription buffer, 8 μL of NTP mix, 2 μL of T7 enzyme mix, and 20 μL of RNase-free water. The mixture was incubated for 2 h at 37 °C. Afterward, additional components were added to bring the total volume to 40 μL, including 1 μL of DNase I, 17 μL of RNase-free water, and 2 μL of RNase T1 (10 U/μL). This solution was incubated for another 30 min at 37 °C. The RNA product was then purified using a magnetic bead-based method.

For the RNAi functional experiment, a total of 30 crayfish were randomly divided into two groups (*n* = 15 per group), one received ds-PcRhoA injections, while the other was administered ds-GFP as a control. Each crayfish was injected with the synthesized dsRNA into the abdominal region [25]. The experimental group received 10 μg of PcRhoA-dsRNA, while the control group was injected with 10 μg of GFP-dsRNA. After 24 h, five individuals from each group had their hepatopancreas, muscles, intestines, and gills collected for total RNA extraction. The remaining crayfish were divided into three groups: one group received 100 μL of Poly I:C at a dosage of 1 μg/g, while the third group was injected with an equivalent volume of PBS as a control. After another 24 h, five individuals from each group were sampled for intestines to extract total RNA. RT-qPCR was performed to assess the silencing efficiency of the *PcRhoA* gene.

### 2.8. Detection of Innate Immunity Signaling Pathway-Associated Genes

To investigate the role of *PcRhoA* in the innate immune system of crayfish, the mRNA expression levels of genes involved in the Toll and Imd signaling pathways were evaluated using RT-qPCR, as described by Zhang et al. (2024) [26]. Following a 24 h treatment with dsRNA, Poly I:C and PBS were injected into the abdominal region of crayfish from the designated groups, which included one group receiving dsRNA targeting the Ferritin Heavy-like gene (dsPcRhoA) and a control group receiving GFP dsRNA (dsGFP). The intestines of five individuals from each group were harvested 24 h after immune challenge. Total RNA was extracted, and cDNA was synthesized. The subsequent analysis of mRNA expression levels of the relevant genes was conducted using RT-qPCR, with a list of the primers used provided in Table 1.

### 2.9. Statistical Analysis

All data are presented as mean ± standard deviation (SD). Data from three separate experiments were combined and analyzed using one-way analysis of variance (ANOVA), followed by a Student’s *t*-test. A *p*-value of less than 0.05 was considered statistically significant [27].

## 3. Results and Discussion

### 3.1. Sequence Analysis of the RhoA Gene

**Figure 1:**
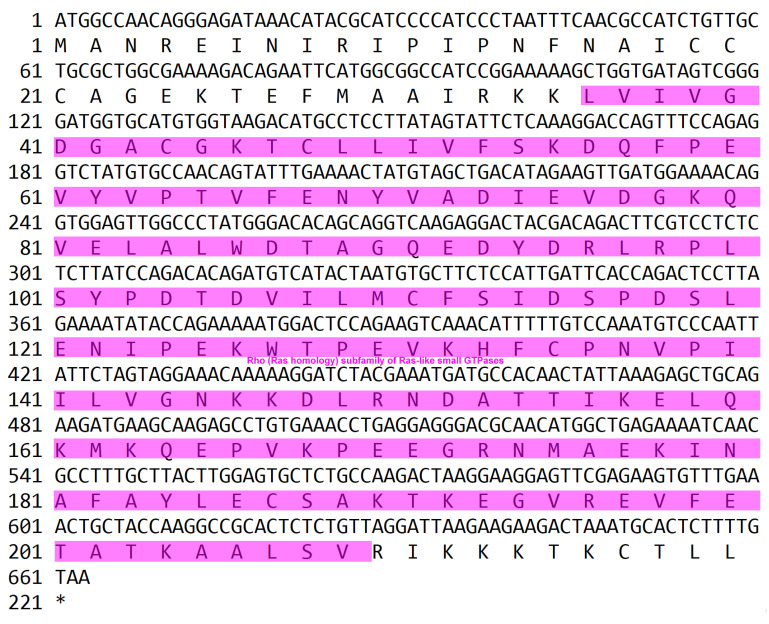
The nucleotide sequence and corresponding deduced amino acid sequence of the PcRhoA gene. The functional domain is highlighted in color.

In our previous transcriptomic analysis [8,18], the full ORF of *PcRhoA* was amplified via PCR from the intestine of *P. clarkii*. The ORF spans 663 bp and encodes a 220-amino-acid polypeptide, which contains a 174-amino-acid Rho-type GTPase domain (GenBank accession number: PX655714) (Figure 1). In silico translation predicted a molecular mass of 24.78 kDa and an isoelectric point of 5.69. Signal peptide screening using the SMART suite identified no N-terminal secretion motif, indicating that PcRhoA functions as an intracellular protein rather than a secreted one.

### 3.2. Homologous Alignment and Phylogenetic Analysis

**Figure 2:**
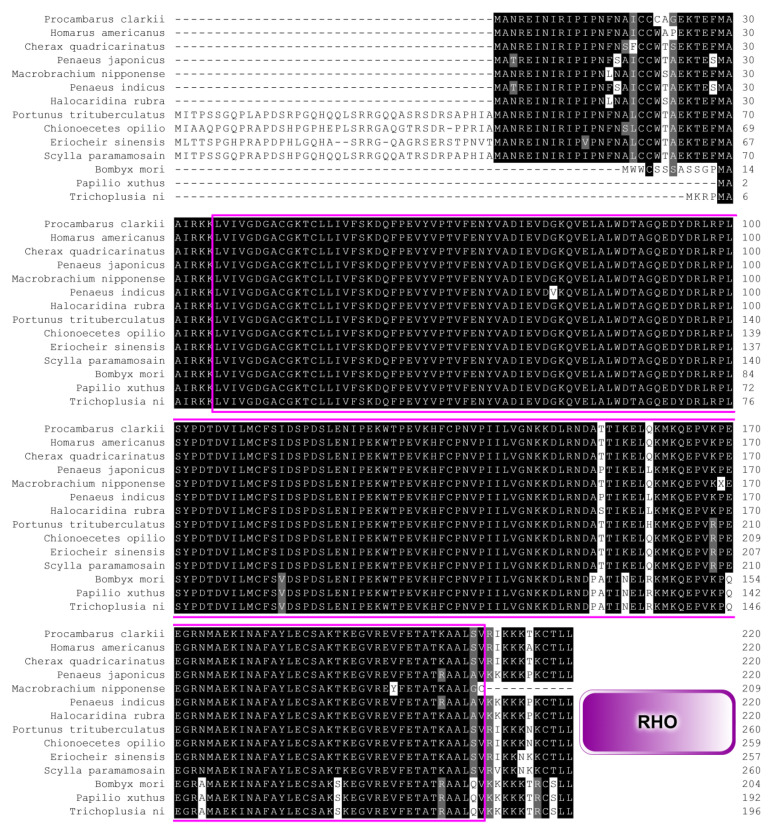
Multiple sequence alignment of the RhoA proteins. The numerical values on the right denote the positional locations of the amino acids across various sequences. The purple border signifies the functional domain associated with RhoA proteins, and the distinct conservation of amino acids is depicted through a variety of colors.

**Figure 3:**
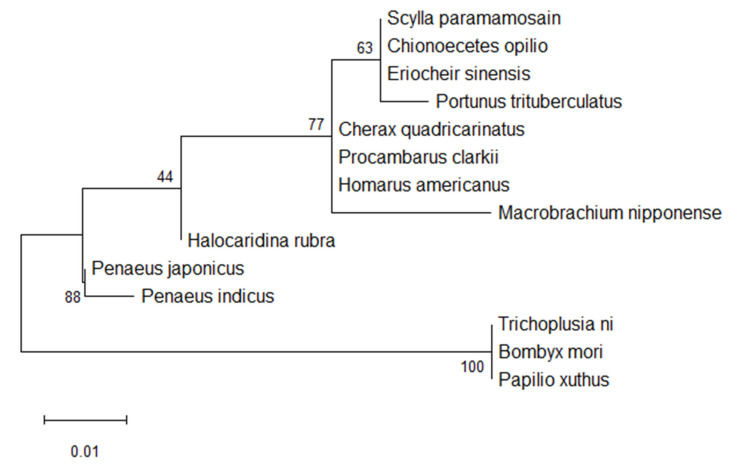
Phylogenetic analysis of RhoA proteins. The tree was constructed from the aligned amino acid sequences using the maximum-likelihood method in MEGA 12. Bootstrap values (from 1000 replicates) are shown at the branch nodes.

To assess the degree of amino acid conservation between PcRhoA and Rho orthologues from other invertebrates, multiple sequence alignment was performed using Clustal X. The alignment revealed extensive identity in the functional domains of *P. clarkii* and closely related taxa, all of which possess the conserved Rho-type GTPase domain (Figure 2). A maximum-likelihood phylogenetic tree was constructed with MEGA 12.0 to investigate the evolutionary relationships among these Rho proteins. As shown in Figure 3, the tree strongly clustered the *P. clarkii* Rho protein with that of *C. quadricarinatus*, suggesting the closest phylogenetic relationship between the two species.

### 3.3. Tissue Distribution of the RhoA Gene

**Figure 4:**
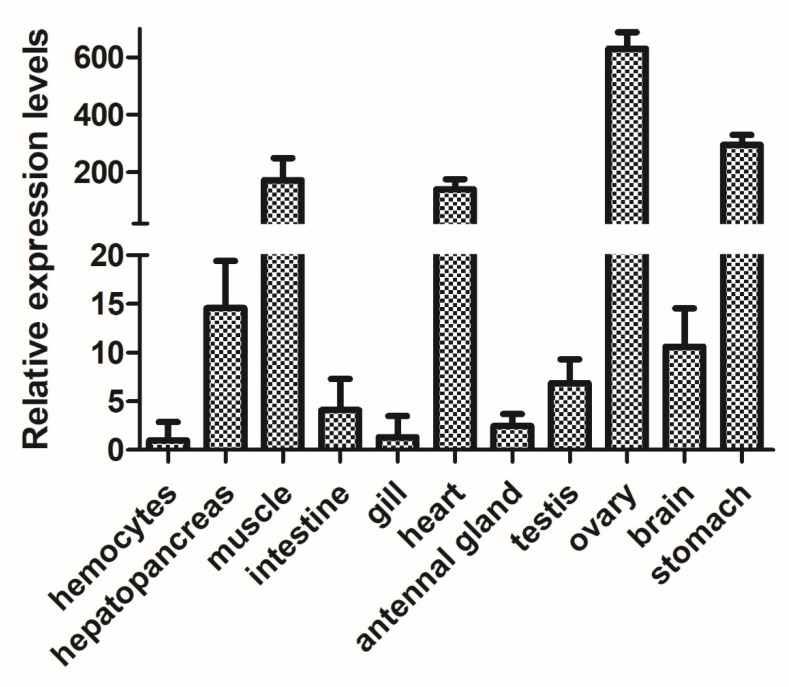
Expression levels of the *PcRhoA* gene in various tissues of normal crayfish. Tissues analyzed include hepatopancreas, muscle, intestine, gill, heart, antennal gland, testis, ovary, stomach, brain, and hemocytes. Gene expression was quantified by RT-qPCR normalized to the *18S* gene as an internal control. Data are presented as mean ± SD (*n* = 5) and were analyzed by one-way ANOVA.

To determine the tissue-specific expression profile of *RhoA*, RT-qPCR was conducted across eleven tissues: hepatopancreas, muscle, intestine, gill, heart, antennal gland, testis, ovary, stomach, brain, and hemocytes. *RhoA* transcripts were detected in all tissues, with significantly higher mRNA levels in the hepatopancreas, muscle, heart, ovary, and stomach. In contrast, relatively low expression was observed in the hemolymph, intestine, gill, and antennal gland (Figure 4).

### 3.4. Quantitative Analysis of RhoA mRNA After Immunity Challenge

**Figure 5:**
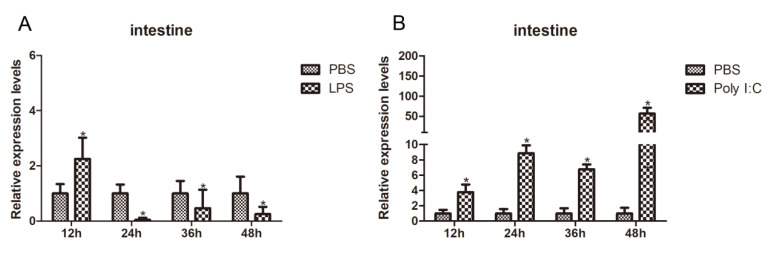
Expression of *PcRhoA* in crayfish intestine following immune challenge. (**A**) Expression profile after LPS injection. (**B**) Expression profile after Poly I:C injection. The PBS-injected group served as the control at each time point. Expression levels were determined by RT-qPCR and normalized to *18S* gene. Data are presented as mean ± SD (*n* = 5). Significant differences compared to the PBS control at the corresponding time point were determined by Student’s t-test and are indicated by asterisks (* *p* < 0.05).

In crustaceans, the intestine functions not only as the primary site for digestion and nutrient absorption but also as a critical immunological barrier against pathogen invasion. This barrier relies on a complex interplay of physical, chemical, and cellular defense mechanisms [28]. To assess the responsiveness of *RhoA* to immune stimuli, its intestinal transcript levels were measured at various time points following LPS (bacterial mimic) and Poly I:C (viral mimic) challenges using RT-qPCR. Compared to the control group, *RhoA* transcripts were significantly upregulated at 12 h post-LPS challenge, peaking before gradually declining (Figure 5A). In response to Poly I:C stimulation, *RhoA* mRNA levels were significantly elevated across all time points sampled (Figure 5B), indicating a heightened sensitivity to viral-like immune challenges. These results confirm that RhoA plays a pivotal role in the intestinal immune defense against pathogen invasion.

### 3.5. RhoA Affects the Transcription of Innate Immunity Signaling Pathway-Associated Genes

**Figure 6:**
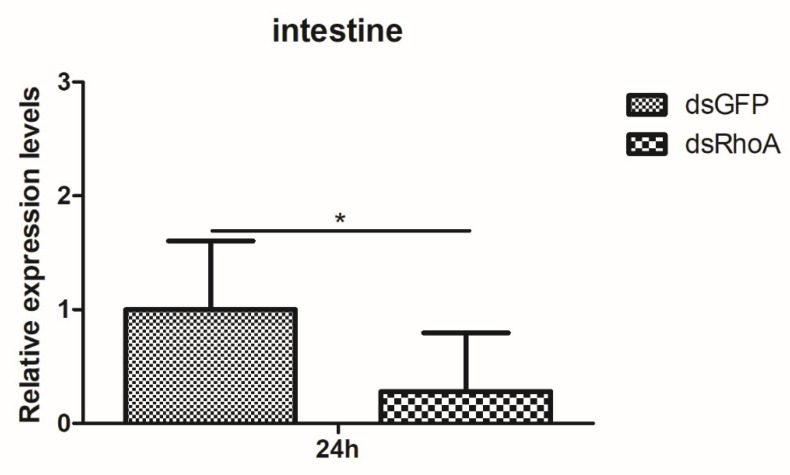
Knockdown of *PcRhoA* gene expression by RNAi. Relative mRNA levels of *PcRhoA* in the intestine of crayfish 24 h after injection with dsRNA targeting *PcRhoA* (*dsRhoA*) or with control dsRNA targeting GFP (*dsGFP*). Gene expression was quantified by RT-qPCR and normalized to 18S. Data are presented as mean ± SD (*n* = 5). Significant differences between the dsRhoA group and the control (dsGFP) group were determined by Student’s t-test and are indicated by asterisks (* *p* < 0.05).

**Figure 7:**
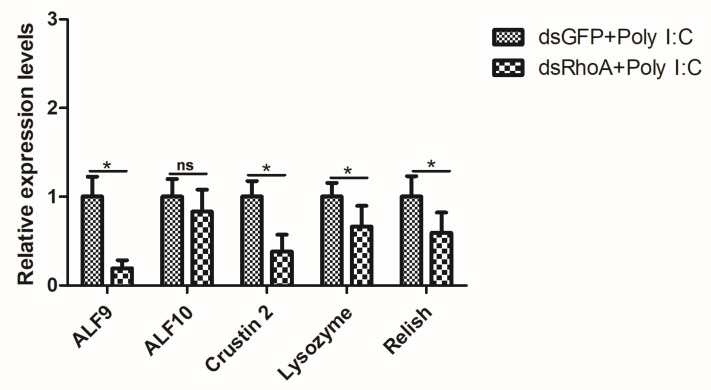
Effect of *PcRhoA* knockdown on the expression of immune-related genes after Poly I:C challenge. Crayfish were injected with PcRhoA-specific dsRNA (dsRhoA) or control dsRNA (dsGFP), followed 24 h later by Poly I:C challenge for 24 h. Relative mRNA levels of the indicated genes (*ALF9*, *ALF10*, *Crustin 2*, *Lysozyme*, *Relish*) in the intestine were analyzed by RT-qPCR. Data are presented as mean ± SD (*n* = 5). The ns represent not significant. Significant differences between the dsRhoA + Poly I:C group and the control (dsGFP + Poly I:C) group were determined by Student’s t-test and are indicated by asterisks (* *p* < 0.05).

RNA interference (RNAi) was employed to functionally validate the role of *RhoA* in the innate immune response of *P. clarkii*. At 24 hpi of dsRNA, total RNA was extracted from the intestines of the three experimental groups to assess RhoA transcript abundance, confirming successful gene silencing (Figure 6). Crayfish were then divided into two treatment groups: one receiving PBS and the other Poly I:C. Total RNA was isolated from intestinal tissues to quantify the mRNA levels of downstream immune signaling genes using RT-qPCR, thereby elucidating the immune function of *RhoA*. Compared to the dsGFP-injected control group, the expression of key downstream effectors—such as anti-LPS factors 9 and 10 (*ALF9* and *ALF10*), crustin 2, lysozyme, and Relish—was significantly downregulated following *RhoA* knockdown and Poly I:C immune challenge (Figure 7). Collectively, these results suggest that *RhoA* may participate in the crayfish innate immune response and could positively influence the transcription of downstream immune effector genes.

## 4. Discussion

The small GTPase superfamily is a critical group of intracellular signaling proteins that regulate cytoskeletal remodeling, cell migration, proliferation, and immune defense [13,29]. RhoA, a prototypical member of this family, has been extensively studied in mammals, where it modulates a wide range of biological processes by regulating actin dynamics and downstream signaling pathways [29]. However, our understanding of RhoA in aquatic organisms, particularly decapod crustaceans, remains limited. It is important to note that the present study provides correlative, transcript-level evidence linking *PcRhoA* to immune processes in *P. clarkii*. While consistent with a potential immune role, the observed expression changes could also reflect broader cellular stress responses or metabolic adjustments to immune challenge. Given the high economic importance of penaeid shrimps and crayfish in global aquaculture, understanding the molecular mechanisms of their immune systems is crucial for improving disease prevention and culture efficiency.

The *RhoA* gene in decapod crustaceans exhibits typical structural features that reflect both its high evolutionary conservation and taxon-specific variations. The gene consists of a protein-coding sequence (CDS) and flanking untranslated regions (5′- and 3′-UTRs). The CDS, usually 600–800 nucleotides long, encodes a polypeptide of approximately 200–260 amino acids, while the UTRs play key roles in transcription initiation, mRNA stability, and translational efficiency. Notably, the 5′- and 3′-UTRs show interspecific variability in length and nucleotide composition among penaeid shrimps and crayfishes, suggesting a role in the species-specific regulation of gene expression [30].

As a core member of the small GTPase family, understanding the evolutionary origin and phylogenetic placement of RhoA in penaeid shrimps and crayfish is essential for deciphering its functional diversity and regulatory mechanisms. Multiple sequence alignments revealed both highly conserved motifs, particularly the RHO-type GTP-binding domain, and lineage-specific substitutions that may underlie physiological traits or adaptations unique to decapod crustaceans. A neighbor-joining phylogenetic tree, based on aligned amino acid sequences, robustly clustered crustacean RhoA proteins into a single, well-supported clade, while insect orthologues formed a distinct, more distant branch. These findings highlight the evolutionary divergence of RhoA within aquatic taxa and mirror the broader phylogenetic separation between Crustacea and Insecta [31].

Establishing a reliable immune-stimulation paradigm is critical for understanding the regulatory role of RhoA in the immune network of aquatic crustaceans [32]. Common immunostimulants include live bacterial or viral inocula, as well as defined pathogen-associated molecular patterns (PAMPs). Bacterial challenges typically use Gram-positive or Gram-negative strains to simulate septic infection, while viral challenges employ aquaculture-relevant viruses or viral mimetics to trigger antiviral responses. PAMPs like LPS and polyinosinic:polycytidylic acid (Poly I:C) are widely used to activate pattern-recognition receptor pathways, replicating natural infection states [33,34]. These approaches effectively prime the crustacean immune system, inducing measurable changes in the expression or activity of signaling molecules, including RhoA, and provide a solid foundation for subsequent mechanistic analysis.

RhoA, a prototypical small GTPase, plays a critical role in the immune responses of decapod crustaceans, coordinating its function through spatio-temporal expression dynamics across immune-relevant tissues. Transcriptomic and RT-qPCR analyses show that bacterial challenges or inflammatory insults rapidly up-regulate *RhoA* expression in haemocytes, intestines, hepatopancreas, and gills, thereby enhancing cytoskeletal remodeling and downstream signaling that amplify innate immunity. Within the inflammatory environment, tumor necrosis factor-α (TNF-α) activates the focal adhesion kinase (FAK)–RhoA axis, triggering ezrin phosphorylation and increasing endothelial permeability, which is vital for immune-cell extravasation and targeted migration. Pathogen-mediated exploitation of this pathway is exemplified by *Listeria monocytogenes*, which induces *RhoA* expression to accelerate macrophage motility and pro-inflammatory cytokine (IL-1β, IL-6, TNF-α) secretion, facilitating translocation across the blood–brain barrier [35].

At the cellular level, *RhoA* acts as a master regulator of actin dynamics, which underpins phagocytosis—the primary defense mechanism by which haemocytes and microglia recognize, engulf, and eliminate foreign particles. Activation of *RhoA* promotes stress-fiber assembly and membrane ruffling, processes that drive pseudopod extension and phagosome formation [36,37]. In contrast, genetic or pharmacological inhibition of *RhoA* impairs macrophage chemotaxis and phagocytic capacity, as demonstrated in spinal-cord injury models where *RhoA* deletion reduces macrophage infiltration and delays tissue repair [38]. Similarly, in a zebrafish spinal transection model, loss of *Kif15* enhanced *RhoA* and *Cdc42* activity, accelerating macrophage migration and efferocytosis, further highlighting the pivotal role of *RhoA* in immune-cell trafficking and functional deployment [39].

*RhoA*, as a central signaling node, integrates multiple immune cascades in aquatic decapods. Through its intrinsic molecular switch activity, *RhoA* orchestrates cytoskeletal remodeling, signal transduction, and gene transcription, bridging pathogen detection to effector responses [40]. TLRs recognize PAMPs and initiate MyD88-dependent pathways that promote the expression of inflammatory cytokines and AMPs. Activated RhoA enhances the assembly of TLR complexes and recruits the adaptor MyD88, amplifying the signal toward NF-κB and MAPK modules. This RhoA-mediated amplification increases microbial recognition efficiency and enhances the migratory and phagocytic capacities of haemocytes. NF-κB, a master transcription factor for immune and inflammatory genes [41,42], is regulated by RhoA, which accelerates IκB kinase (IKK) complex activity, leading to IκB phosphorylation and degradation, thereby enabling NF-κB nuclear translocation. In crayfish challenged with bacteria, RhoA activity coincides with NF-κB activation, driving the elevated transcription of AMPs and pro-inflammatory cytokines. Additionally, RhoA-driven actin dynamics may influence the spatiotemporal distribution of NF-κB signaling, fine-tuning the intensity and duration of the immune response [43]. The RhoA-ROCK signaling pathway intersects with the MAPK cascade (ERK, JNK, p38), modulating cell proliferation, stress responses, and cytokine production. In crustaceans, the RhoA-ROCK axis cooperates with MAPK pathways to potentiate pro-inflammatory cytokine release and haemocyte activation, ensuring rapid and robust defense against pathogens [44].

Transcriptomic profiling of *Macrobrachium rosenbergii* infected with *Aeromonas dhakensis* revealed a coordinated up-regulation of *RhoA* and immune effectors such as CASP9 and PKC, suggesting a shared regulation of apoptosis and immunity [45]. In contrast, *Vibrio* spp., among the most destructive bacterial pathogens in shrimp aquaculture, exploit RhoA-driven cytoskeletal rearrangements to facilitate invasion and dissemination [46]. While this study provides transcriptional evidence linking *PcRhoA* to immune regulation in *P. clarkii*, several limitations should be noted. First, our conclusions are based solely on mRNA expression data; protein-level changes and post-translational activation of RhoA were not assessed. Second, functional validation relied exclusively on Poly I:C challenge, and no direct bacterial or viral pathogen challenges were conducted to evaluate organism-level outcomes such as survival or pathogen clearance. Third, although downstream immune genes were examined, direct pathway activity assays (Toll, IMD, NF-κB or MAPK activation) were not performed. Therefore, while our data are consistent with a role for *PcRhoA* in innate immunity, further mechanistic and physiological studies are needed to confirm its precise function. Thus, understanding *RhoA*-centered signaling networks is critical for developing molecular strategies to mitigate Vibrio-induced losses. *RhoA* is potentially involved in modulating both immune activation and apoptotic processes, providing flexibility and adaptability to the crustacean immune system. Dissecting this balance will reveal novel molecular targets for precision immunomodulation, offering innovative approaches to sustainable disease control in aquaculture.

## 5. Conclusions

The cloning and characterization of *PcRhoA*, the red swamp crayfish orthologue of *RhoA*, provide preliminary evidence that this small GTPase may be involved in innate immunity. *PcRhoA* is predominantly expressed in immune-relevant tissues and is rapidly up-regulated in the intestine following LPS or Poly(I:C) challenges. Silencing *PcRhoA* was associated with the suppression of downstream immune effectors, suggesting its potential role in immune signal transduction. These findings identify *PcRhoA* as a candidate immune-related gene and highlight a potential molecular target worthy of further investigation for enhancing disease resistance in aquaculture.

## Figures and Tables

**Table 1:** Primers used in this study.

Primer Name	Primer Sequences (5′-3′)	Tm (°C)	Size (bp)
PcRhoA-F	ATGGCCAACAGGGAGATAAAC	58	623
PcRhoA-R	TTACAAAAGAGTCATTTAGTCT		
Pc18S-F	ACCGATTGAATGATTTAGTGAG	55	119
Pc18S-R	TACGGAAACCTTGTTACGAC		
PcRhoA-Fi	GCGTAATACGACTCACTATAGGGAAAGACAGAATTCATGGCGG	57	446
PcRhoA-Ri	GCGTAATACGACTCACTATAGGGGTCCCTCCTCAGGTTTCACA		
GFP-Fi	GCGTAATACGACTCACTATAGGTGGTCCCAATTCTCGTGGAAC	57	428
GFP-Ri	GCGTAATACGACTCACTATAGGCTTGAAGTTGACCTTGATGCC		
PcRhoA-RT-F	AACAGGGAGATAAACATACGC	56	170
PcRhoA-RT-R	GGAAACTGGTCCTTTGAGAAT		
PcALF9-RT-F	GTCGGGCTGTTTAGGAATGAGG	55	145
PcALF9-RT-R	TTGTCTTGTTCGCCACTCCACTT		
PcALF10-RT-F	TGTCTGCTCTTTGCTCGTTC	55	169
PcALF10-RT-R	GTGTCGTCAATAGATACTGCGTTA		
Pccrustin2-RT-F	CTGGTGTTGTCCATGCTGGTG	55	171
Pccrustin2-RT-R	CCTGAGGTGGTAGGATTCTTGT		
Pclysozyme-RT-F	AGCCCTCGTGGTCGTCTTG	55	186
Pclysozyme-RT-R	GTTGGGATCGGCGTTATTG		
PcRelish-RT-F	GCTGTCCGTGGCAATGAAG	55	138
PcRelish-RT-R	GAGGCAGTGCTGAACGAGTG		

## Data Availability

The datasets generated for this study can be found in the GenBank accession no. PX655714.
